# Effects of desensitizing products on the reduction of pain sensitivity caused by in-office tooth bleaching: a 24-week follow-up

**DOI:** 10.1590/1678-7757-2019-0755

**Published:** 2020-06-24

**Authors:** Josué Junior Araujo PIEROTE, Lucia Trazzi PRIETO, Carlos Tadeu dos Santos DIAS, João Victor Frazão CÂMARA, Débora Alves Nunes Leite LIMA, Flávio Henrique Baggio AGUIAR, Luis Alexandre Maffei Sartini PAULILLO

**Affiliations:** 1 Universidade Estadual de Campinas Faculdade de Odontologia de Piracicaba Departamento de Dentística Restauradora Piracicaba Brasil Universidade Estadual de Campinas, Faculdade de Odontologia de Piracicaba, Departamento de Dentística Restauradora, Piracicaba, Brasil.; 2 Universidade de São Paulo Escola Superior de Agricultura Luiz de Queiroz Departamento de Engenharia Agronômica Piracicaba Brasil Universidade de São Paulo, Escola Superior de Agricultura Luiz de Queiroz, Departamento de Engenharia Agronômica, Piracicaba, Brasil.; 3 Universidade de São Paulo Faculdade de Odontologia de Bauru Departamento de Ciências Biológicas Bauru Brasil Universidade de São Paulo, Faculdade de Odontologia de Bauru, Departamento de Ciências Biológicas, Bauru, Brasil.

**Keywords:** Tooth bleaching, Dentifrices, Fluoride, Sensitivity

## Abstract

**Objective:**

To clinically assess the effect of desensitizing gels and dentifrices on the reduction in pain sensitivity and color variation during tooth bleaching.

**Methodology:**

A total of 108 volunteers were randomly separated into the following groups of n=12: GT/S-glycerine and thickener/sucralose; NF/S-potassium nitrate and sodium fluoride/sucralose; NA/S-potassium nitrate and arginine/sucralose; GT/AC-glycerine and thickener/arginine and calcium carbonate; NF/AC-potassium nitrate and sodium fluoride/arginine and calcium carbonate; NA/AC-potassium nitrate and arginine/arginine and calcium carbonate; GT/PN-glycerine and thickener/potassium nitrate; NF/PN-potassium nitrate and sodium fluoride/potassium nitrate; and NA/PN-potassium nitrate and arginine/potassium nitrate. Sensitivity was assessed with the numerical analogue scale, and color variation (ΔE) was measured with a spectrophotometer. The sensitivity values obtained were subjected to a multivariate analysis of variance (MANOVA) and color variation values were subjected to a randomized analysis of variance (p<0.05).

**Results:**

The NF/AC, NA/AC, NF/PN, and NA/PN groups presented lower sensitivity values and reduced sensitivity compared to those of the other groups throughout the clinical sessions. None of the groups showed sensitivity at the 24-week assessment. Statistically, no significant difference were observed in the color values among the groups four weeks after the beginning of bleaching (p=0.074). Additionally, the color assessment of all groups was statistically similar four weeks (p=0.084) and 24 weeks (p=0.118) after the beginning.

**Conclusion:**

Our results indicate that adding NF/S, NA/S, NF/AC, and NA/AC desensitizers to tooth bleaching protocols reduces pain sensitivity without affecting its effectiveness.

## Introduction

Sensitivity is the main adverse effect of tooth bleaching;^1-3^it is reported by at least two thirds of patients,^4,5^ and it occurs mainly in the first weeks of the treatment.^6,7^ The etiology of this symptom has been attributed to the amount of hydrogen peroxide that reaches the pulp. The perception of pain is a consequence of the activation of the receptors sensitive to neuronal peroxide, thus generating a neuro-response of the afferent sensory endings of the nerve fibers.^4,5,8,9,10^

Some clinical techniques used to mitigate this side effect include reducing the concentration and application time of hydrogen peroxide, decreasing the frequency of bleaching gel applications,^4,5^ administering analgesic/anti-inflammatory treatments,^11^ and using desensitizers.^1,6,7,12^

Desensitizing agents work via two action mechanisms. One mechanism involves the use of agents such as fluoride and arginine to obliterate the dentinal tubules, preventing the movement of dentinal fluids and assisting the remineralization of dentin.^1,5,13^ The other mechanism blocks the activity of the pulp nerve, decreasing the sensory excitability of nociceptors,^1,5^ which often involves agents such as potassium nitrate.

These desensitizing agents can be applied in the dental office before/after the bleaching treatment or can be self-administered by the patient at home using specific dentifrices and fluoride gels.^14^ The literature shows that the topical application of 5% potassium nitrate with 2% sodium fluoride before the bleaching gel reduced sensitivity during the treatment; however, it was not effective in the interval between the sessions.^14-16^ Thus, the use of such products could not eliminate definitively bleaching-related sensitivity.^17^ Additionally, there is a lack of assessment of potassium nitrate combined with other desensitizing products, such as bioglass and arginine, which have been used in desensitizing dentifrices and that can improve the effects of potassium nitrate when used as desensitizing agents.^14,15,18^

*In vitro* dentifrices containing bioglass or arginine applied before tooth bleaching were effective in protecting enamel against the mineral loss promoted by bleaching without interfering with treatment results.^18^ Some studies have addressed the effect of applying desensitizing dentifrices on tooth sensitivity caused by bleaching.^16^ However, there are no studies formulating and assessing the effect of desensitizers containing arginine on dentin sensitivity.^19,20^ Therefore, the development of desensitizers containing arginine and potassium nitrate that affect tooth sensitivity caused by tooth bleaching, as well as their longitudinal clinical assessment, is necessary.

Our study aimed to compare the clinical performance of an experimental and commercial desensitizing agent and its combination with commercial dentifrices. The desensitizing performance was measured by the reduction in pain sensitivity and color variation during in-office tooth bleaching. The commercial dentifrices tested were assessed longitudinally through a double-blind controlled clinical trial. The null hypothesis was that the use of experimental and commercial desensitizers combined with desensitizing dentifrices would not interfere with the bleaching color variation.

## Methodology

### Ethical Aspects

This study was conducted in full accordance with the World Medical Association Declaration of Helsinki.^21^ The project was submitted to and approved by the Research Ethics Committee affiliated with the National Commission of Research Ethics (CONEP) under protocol number 104/2015 and registered in the Clinical Trials Records and Results Database (ClinicalTrials.gov) under protocol NCT03019224. All volunteers signed a consent form. The clinical trial was reported according to the standard protocol of the CONSORT statement.^22^

### Material

**Figure 1:**
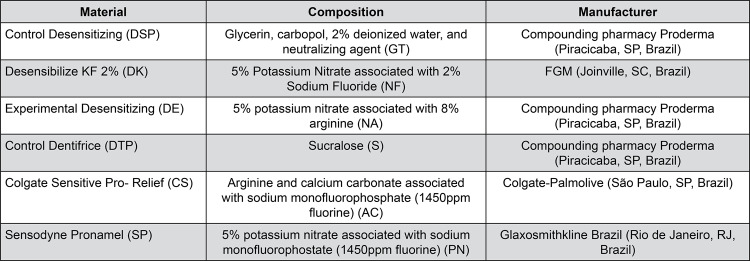
Description of the desensitizing products, their composition and manufacturers

Three types of desensitizing gels and three types of dentifrices were used in the assessment of desensitizing products (Figure 1).

### Experimental Design

A longitudinal double-blind controlled clinical trial was performed with 108 volunteers. The objects of study included a desensitizing gel and a desensitizing dentifrice, investigated at three levels: two levels for treatment and one level for control. The response variables included pain sensitivity and color variation (ΔE).

### Experimental Groups

**Figure 2:**
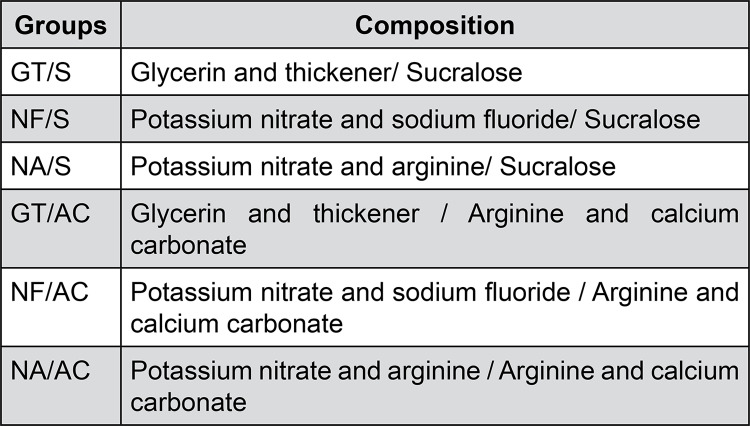
Groups and their active ingredients used in the experiment.

The interaction between the desensitizer and the dentifrice resulted in nine groups (Figure 2).

### Clinical Procedures

#### a) Selection and preparation of volunteers

We invited patients that attended an undergraduate clinic for bleaching treatment to participate in the study. The researcher, who did not participate in the randomization process, informed the volunteers about all aspects of the study, emphasizing that they were free to refuse to participate, withdraw their consent, or even stop participating at any time. Additionally, the patients were explained that their participation was voluntary and that their refusal to participate would not result in any penalty or loss of benefits related to the treatment.

The participants signed an informed consent form, and the initial clinical assessment was applied after they read the study information carefully.

The inclusion criteria were age between 18 and 30 years, good oral and general health, healthy anterior teeth with chroma superior to A2 on the Vita Classical color scale (VITA Zahnfabrink, Bad Säckingen, Germany), the submission of a signed consent form, and availability for all the in-person visits.

The exclusion criteria were being a smoker, being a pregnant or lactating woman, having undergone previous tooth bleaching, currently use of desensitizers or orthodontic appliances, engaging in parafunctional habits, experiencing dentin sensitivity, having gingival recession, having non-carious cervical lesions, having anterior teeth with restorations and carious lesions, having experienced nonvital tooth darkening, and having unsatisfactory posterior restorations.

The volunteers were assessed through anamnesis and clinical examination with a clinical mirror and dental explorer. Interproximal and periapical radiographs were taken for the radiographic examination. This assessment allowed to determinate if patients met the inclusion criteria, which resulted in a sample of 108 volunteers.

After the selection of volunteers, oral conditioning was performed by supragingival scaling with periodontal curettes, and prophylaxis was performed with rubber cups at a low rotation using water/pumice paste.

One week before the beginning, a specific toothpaste (Colgate Total 12, Colgate-Palmolive, São Paulo, Brazil) and a toothbrush (Slim Soft, Colgate-Palmolive, São Paulo, Brazil) were given to the volunteers, followed by recommendations for their use for oral hygiene until they started the bleaching sessions.

#### b) Randomization

**Figure 3:**
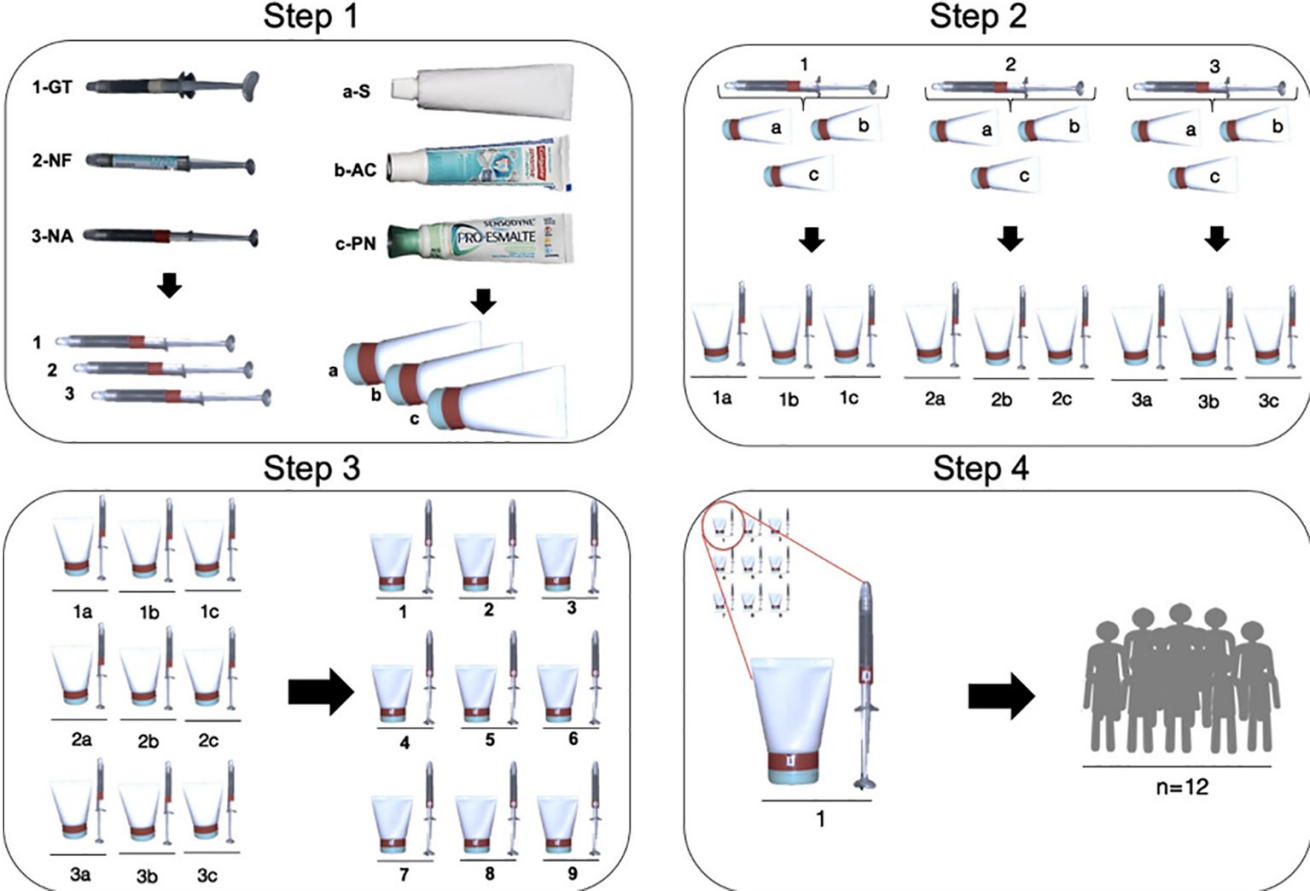
Randomization of the study

After the selection of the volunteers, a researcher (responsible for steps 1, 2, and 3) and a dentist (responsible for step 4 and the clinical stages) randomized the groups. Step 1 consisted of removing desensitizers and dentifrices from the original packaging; step 2, of combining the products; step 3, of coding the respective combination and Step 4, of desensitizing and dentifrices distribution, as shown in Figure 3.

#### c) Use of desensitizing dentifrices

Between the clinical bleaching sessions, each volunteer used an unidentified dentifrice corresponding to the experimental group, which was previously selected by a professional, who did not participate in the study. Thus, both the researcher (dentist) that provided the dentifrice and the volunteer were not aware of the experimental group to which the volunteer belonged (double blind).

After the first in-office bleaching session, volunteers were instructed to use only the specific dentifrice provided according to their group and in a small amount (two thirds of the toothbrush area) three times a day (after breakfast, after lunch, and after dinner) until one week after the end of the third bleaching session.

#### d) Colour assessment

An objective assessment was performed using a spectrophotometer (Vita Easyshade Advance, Vident, Brea, CA, USA) before the first bleaching session, one week after the end of bleaching and 24 weeks after the beginning of the bleaching treatment.

The color was always assessed in the same position through a silicone guide on hydrated teeth. For the preparation of the guide, an impression of the upper incisors was obtained using addition silicone (Express XT Pasta Densa Soft, 3M ESPE, Sumaré, SP, Brazil). Tooth color was assessed through an opening compatible with the tip size of the spectrophotometer, in the middle third on the buccal surface of the right upper central incisor of the guide.

The color was determined using the parameters of the Easyshade device, which indicates the following values: L*, a*, and b*. The L* parameter represents the tooth value on a scale from 0 (black) to 100 (white), and the a* and b* parameters represent the shadow, in which a* is the measure along the red (a* positive) and green (a* negative) axes and b* is the measure along the yellow (b* positive) and blue (b* negative) axes. The color was compared using color variation (ΔE), being estimated with the equation ΔE = ((ΔL*)2+(Δa*)2+(Δb*)2)1/2. This evaluation was performed to verify the effectiveness of the bleaching treatment and if the dentifrices used would affect the quality of the treatment.

#### e) Isolation of the surgical field

The clinical procedures were performed under relative isolation using a lip retractor (Arcflex, FGM, Joinville, SC, Brazil) and cotton rolls to create a gingival barrier, a desensitizing agent, and a bleaching agent from the right first molar to the left first molar of the upper and lower arches.

#### f) Application of the gingival barrier

The gingival barrier (Top Dam, FGM, Joinville, SC, Brazil), approximately 3 mm high, was inserted in the gingival margins and the papillae of the teeth that received the bleaching gel. Groups of three teeth each were then photopolymerized for 20 seconds using high power LEDs (irradiance = 600 MW/cm^2^) (RadiiCal, Sao Paulo, SP, Brazil).

#### g) Application of the desensitizing agent

As previously mentioned, the specific desensitizing gel was previously selected by a professional that did not participate in the study. Thus, both the researcher (dentist) that provided the dentifrice and the volunteer were not aware of the experimental group to which the patient belonged (double blind).

The desensitizing gel was applied with a microbrush applicator. The product remained on the buccal surface of all teeth from the right first molar to the left first molar of the upper and lower arches for 10 minutes. Then, the desensitizer was removed with a water spray and a disposable plastic suction cannula.

#### h) Application of the bleaching agent

The hydrogen peroxide was prepared by mixing 24 drops of 35% hydrogen peroxide (Whiteness HP, FGM, Joinville, SC, Brazil) with 8 drops of thickener, according to the manufacturer’s recommendations. The bleaching agent needs to be rubbed on the tooth surface (3-4 times per application) to remove the oxygen bubbles and to improve the contact between the gel and the teeth. The gel remained in contact with the buccal surface of the teeth for 15 minutes, being removed with a suction cannula and water spray. This procedure was performed three times per clinical session. The volunteers underwent three clinical bleaching sessions, weekly.

#### i) Assessment of pain sensitivity

Sensitivity was assessed seven times as follows: S1, immediately after the first session; S2, 24 hours after the first session; S3, immediately after the second session; S4, 24 hours after the second session; S5, immediately after the third session; S6, 24 hours after the third session; and S7, 24 weeks after the first bleaching session. To assess pain sensitivity, the numerical rating scale was applied at the dental office, with scores ranging from 0 to 10.

## Statistical Analysis

The pain sensitivity data were subjected to multivariate analysis of variance (MANOVA) with repeated measures and a Lambda Wilks test (p<0.05). For the color variation analysis, a completely random analysis of variance was applied (p<0.05).

## Results

**Figure 4:**
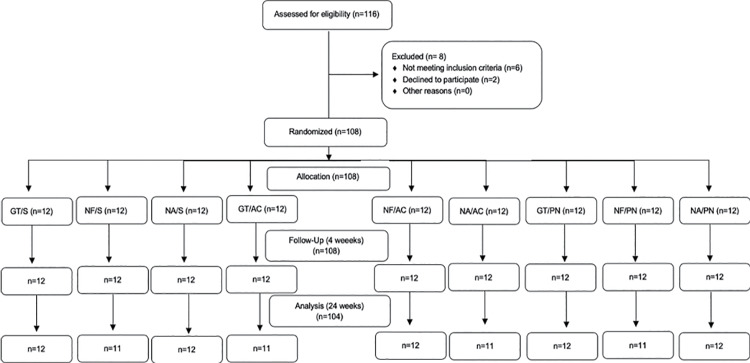
CONSORT flow chart

After four weeks, 108 participants were still participating in the study (50 men and 58 women), and after 24 weeks, 104 participants completed the study (50 men and 54 women) (Figure 4).

**Figure 5:**
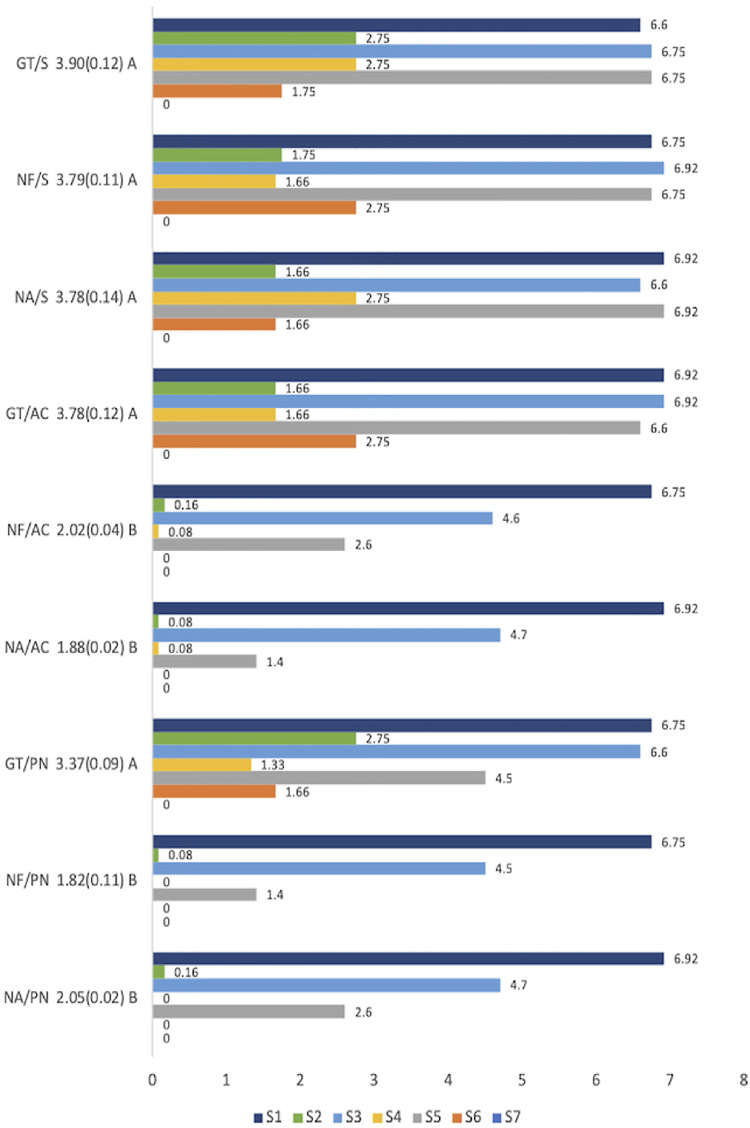
Mean, standard deviation, and Lambda Wilks test for pain sensitivity during the bleaching treatment

After four weeks, the means of the sensitivity values among the groups were compared two by two, and the results showed a significant difference (p<0.05) between the groups (Figure 5). Additionally, the groups showed no sensitivity at the 24-week assessment. The comparison among the groups after 24 weeks of the beginning of the bleaching treatment showed (p=1) no significant differences (p≥0.05).

Figure 5 shows the pain sensitivity of each group in the seven assessments. The NF/AC (2.02), NA/AC (1.88), NF/PN (1.82), and NA/PN (2.05) groups showed the lowest pain sensitivity levels among all groups (GT/S (3.90). The NF/S (3.79), NA/S (3.78), GT/AC (3.78), and GT/PN (3.37) showed the highest pain sensitivity.

Figure 5 shows that, for the analysis of sensitivity over time, the same groups that presented the lowest levels (NF/AC, NA/AC, NF/PN, and NA/PN) also presented a sensitivity reduction throughout the clinical bleaching sessions. Additionally, no groups reported sensitivity at the 24-week assessment.

The color variation was visually perceptible after four weeks (ΔE> 3.3)18 and after 24 weeks (Table 1). Complete random analysis of variance was used to evaluate color variation, and the values after 24 weeks were lower than the values after four weeks, without a significant difference between them (p=0.074) (Table 1). Additionally, the comparison among groups showed statistical similarity at the four-week (p=0.084) and 24-week (p=0.118) assessments after in-office tooth bleaching (Table 1).

**Table 1:** Tukey’s test results for ΔE values and their parameters after four and twenty four weeks

Groups	ΔE 4w	ΔE 24w	ΔL 4w	ΔL 24w	ΔL 4w	ΔL 24w	ΔL 4w	ΔL 24w
	Mean (SD)	Mean (SD)	Mean (SD)	Mean (SD)	Mean (SD)	Mean (SD)	Mean (SD)	Mean (SD)
GT/S	4.91 (1.06)^aA^	3.40 (0.94)^aA^	1.21 (0.06)^bB^	1.82 (0.08)^bB^	0.91 (0.06)^cC^	0.88 (0.06)^bB^	-0.62 (0.06)^dD^	-0.83 (0.06)^dD^
NF/S	4.61 (1.19)^aA^	3.56 (1.02)^aA^	0.91 (0.19)^bB^	1.16 (0.19)^bB^	0.61 (0.19)^bB^	0.72 (0.15)^bB^	-0.81 (0.19)^dD^	-0.74 (0.20)^dD^
NA/S	4.51 (1.20)^aA^	3.37 (0.83)^aA^	0.71 (0.20)^bB^	1.42 (0.31)^bB^	0.51 (0.10)^bB^	0.62 (0.13)^bB^	-0.83 (0.20)^dD^	-0.63 (0.10)^dD^
GT/AC	4.42 (1.49)^aA^	3.39 (1.05)^aA^	1.02 (0.49)^bB^	1.02 (0.49)^bB^	0.82 (0.49)^bB^	0.74 (0.41)^bB^	-0.62 (0.49)^dD^	-0.42 (0.09)^dD^
NF/AC	4.57 (1.31)^aA^	3.64 (1.02)^aA^	0.90 (0.31)^bB^	1.07 (0.31)^bB^	0.57 (0.21)^bB^	0.68 (0.24)^bB^	-0.86 (0.31)^dD^	-0.76 (0.09)^dD^
NA/AC	4.81 (1.81)^aA^	3.41 (1.02)^aA^	1.01 (0.61)^bB^	1.09 (0.91)^bB^	0.81 (0.12)^bB^	0.92 (0.32)^bB^	-0.81 (0.21)^dD^	-0.64 (0.13)^dD^

*Coefficients with the same letter show no statistical difference among the means of color variation.

*Lower-case letters compare the rows.

*Capital letters compare the columns.

## Discussion

As expected, the hypothesis that the use of experimental and commercial desensitizers combined with desensitizing dentifrices reduces tooth sensitivity without interfering with color variation was confirmed.

The sensitivity values showed that the group that used products containing glycerine and thickener or sucralose (GT/S) showed no significant difference when compared with NF/S, NA/S, GT/AC, and GT/PN groups. These products share the same glycerine and thickener or sucralose with the addition of potassium nitrate and arginine or calcium carbonate. However, groups that used products lacking glycerine and thickener or sucralose (NF/AC, NA/AC, NF/PN, and NA/PN) presented a significant reduction in sensitivity compared to that of the other groups.

Glycerine is the commercial form of glycerol, which is simply a colorless, viscous, and sweet-tasting liquid organic compound that has been used because its viscosity is similar to that of commercial desensitizers and does not affect sensitivity.^20^ Despite being made of and taste like sugar, our body does not recognize sucralose as a carbohydrate; and it has zero calories. Hence, it is not used as a substrate for the oral bacteria that cause caries, not affecting tooth sensitivity, which justifies its use in the control dentifrice.^23^ These two compounds were used as placebos (controls), given the double-blind design of the study.

The NF and NA desensitizers combined with the S control dentifrice did not reduce sensitivity (NF/S and NA/S), because such compounds require more time in enamel to show effectiveness. The application time of the desensitizing gel may not have been enough for NF and NA to diffuse into the enamel, obliterate the dentinal tubules, and facilitate the movement of fluids through enamel and dentin.^23^ This is consistent with the findings that products containing potassium nitrate combined with fluoride or arginine at small concentrations are only able to reduce dentin sensitivity near the fourth week of use.^23,24^ The action of NF and NA was even more limited in enamel, as shown in our study.

Regarding the NA experimental desensitizer, even with potassium nitrate in its composition, the obliteration of dentinal tubules by arginine did not allow a reduction in the excitability of the nerve fibers through the inhibition of sodium and potassium ion movements around sensory fibers, which are usually promoted by potassium nitrate.^25,26^

The NF/AC, NA/AC, NF/PN, and NA/PN groups showed a significant reduction in pain sensitivity after using combinations of desensitizers containing NF or NA with dentifrices containing AC or PN (p<0.05). This result is justified by the synergic action of arginine and potassium nitrate when combined with desensitizing dentifrices.^25,26^

The NA desensitizer and the AC dentifrice can diffuse through the enamel, being deposited on dentin surfaces to physically block and seal the dentinal tubules.^25,26^This technology, based on arginine-containing products, promotes diffusion through prisms of bleached enamel, physically obliterating and forming a plug in the dentinal tubules, thus allowing relief of pain sensitivity.^25-27^ This new technology provides clinically proven benefits over rapid and long-lasting sensitivity relief. It also shows that arginine works to accelerate the natural tubular occlusion mechanisms with a protective layer deposited on the surface of the dentin adjacent to the bleached enamel.^28^ Clinical findings show that arginine-containing products provide significant sensitivity relief.^25,26^

The NF desensitizers and PN dentifrices block the sensory activity of the nerve fibers of the pulp and decrease the sensory excitability of nociceptors.^4,28-30^ Potassium nitrate diffuses through enamel and dentin into the nerve endings of sensory fibers, reducing the excitability of nerve fibers by inhibiting the movement of sodium and potassium ions around the sensory fibers. This action results in the modulation or suppression of the painful sensation.^24,31,32^ Duo to this mechanism, potassium salts have been suggested as an effective treatment for pain sensitivity caused by tooth bleaching.^4,24^ Our study shows that products with nitrate may be more effective than fluoride in reducing pain after tooth bleaching, supporting results described in previous studies.^16,32^

A reduction in sensitivity during tooth bleaching is beneficial because it provides a better comfort in the procedure, which facilitates the patient’s commitment to treatment.^4,16^ The combination of dentifrices and desensitizers in our study proved to be efficient in reducing pain sensitivity caused by in-office tooth bleaching.

The sensitivity assessment showed that sensitivity values 24 hours after applying the bleaching agent in the last two sessions decreased significantly in the groups that used dentifrices containing AC or PN (NF/AC, NA/AC, NF/PN, and NA/PN). This was due to the continued use of the dentifrice, which allowed a longer contact between the toothpaste and the dental surface, inhibiting painful symptomatology.^16,24,32^

The combination of desensitizers with dentifrices did not affect the results of the bleaching treatment, considering that there was no significant difference in tooth shades among the groups evaluated. Desensitizers containing NA and dentifrices containing AC were expected to affect the diffusion of the bleaching gel due to their mechanism of action, which is similar to that of fluoride, as both promote a reduction in enamel permeability and the obliteration of dentinal tubules. Nevertheless, the hydrogen peroxide molecule is relatively small and can penetrate the spaces between the enamel prisms.^33,34,35^This probably explains the similar results of color variation after bleaching among the groups. Then, the first null hypothesis that experimental and commercial desensitizers combined with desensitizing dentifrices would not interfere with the bleaching color variation was confirmed.

Our study showed that the combination of desensitizers containing NF or NA with dentifrices containing AC or PN might be an efficient alternative to reduce pain sensitivity caused by in-office tooth bleaching. However, the limitations of this study are related to the need for a long-term follow-up of volunteers to analyze color stability after the end of the bleaching treatment. Consequently, we suggest further studies with longer follow-up periods.

## Conclusion

The use of desensitizers containing potassium nitrate and sodium fluoride or potassium nitrate and arginine combined with dentifrices containing arginine and carbonate or potassium nitrate represent a viable technique, since they reduce pain sensitivity during in-office tooth bleaching sessions without interfere with treatment result.
